# Squirting Scripts: Navigating Validation, Avoidance, Desire, and Coercion in Sexual Encounters Between Women and Men

**DOI:** 10.1007/s10508-026-03425-1

**Published:** 2026-04-08

**Authors:** Jessica Påfs

**Affiliations:** 1 Department of Social Work, University of Gothenburg, Box 720, 405 30 Gothenburg, Sweden; 2 Experience Lab, Department of Social Sciences, Åbo Akademi University, Vaasa, Finland; 3 Department of Psychology, Åbo Akademi University, Turku, Finland

**Keywords:** Female ejaculation, Squirting, Sexual pleasure, Orgasm, Sexual script, Gender

## Abstract

Female ejaculation, commonly referred to as squirting, is the emission of fluid during sexual arousal or orgasm. Although increasingly recognized as a relatively common experience, scientific debate continues regarding the nature and origin of the expelled fluid. Furthermore, squirting remains underexplored in relation to women’s own perspectives, particularly concerning the interpersonal negotiation and partners’ responses. This study investigated cisgender women’s experiences of responses from men as sexual partners to squirting, aiming to conceptualize the interpersonal meanings of squirting within sexual interactions between women and men, examined through the notion of “squirting scripts.” Data were collected through in-depth interviews in Sweden. The analysis identified four key themes: (1) Validated: Squirting as a neutral bodily response, met with supportive or accepting responses; (2) Avoided: Squirting as an undesirable sexual response, accompanied by negative or dismissive partner responses; (3) Desired: Squirting as tangible proof of sexual prowess, where partners expressed pride and viewed it as an achievement or confirmation of climax—sometimes in contrast to the woman’s own experience; and (4) Coerced: Squirting as a pressured performance, where women felt pushed beyond their boundaries to satisfy a partner’s desire for this response. These findings reveal that squirting is imbued with conflicting meanings—ranging from neutral acceptance and pride to discomfort and coercion—influenced by the responses of men as partners and cultural expectations. While some women described feeling affirmed or empowered, others recounted navigating shame, avoidance, or pressure to perform. Squirting in sexual interactions between women and men thus emerges not as a purely physiological event, but as a socially charged experience, negotiated and expressed through diverse squirting scripts.

## Introduction

Female ejaculation, commonly referred to as squirting, is the emission of fluid during sexual arousal or orgasm, a phenomenon also previously known as female orgasmic expulsion or gushing (Addiego et al., 1981; Belzer et al., 1984; Rubio-Casillas & Jannini, 2011; Salama et al., 2015; Sevely & Bennett, 1978). Scientific literature has often characterized squirting as a unique rarity, yet recent research, including a first-ever prevalence study, indicates it is a common experience, with 41% of women reporting having squirted (Hensel et al., 2024). Similar percentages have been reported in convenience samples from the USA (54%), North America (40%), and Egypt (40%) (Darling et al., 1990, 1991; Younis et al., 2015). Despite its prevalence, recent qualitative studies suggest many women are unaware of squirting before their own experience and advocate for better information on the subject (Gilliland, 2009; Påfs, 2023).

### Physiological Ambiguity and Definitional Discord

Physiological understanding of the fluid’s makeup and origin remains constrained, with claims it is female prostatic fluid, diluted urine, or a fluid identical to urine (Addiego et al., 1981; Inoue, 2022; Pastor, 2013; Rubio-Casillas & Jannini, 2011; Salama et al., 2015; Sevely & Bennett, 1978). This ambiguity recently led to attempts to distinguish female ejaculation and squirting as two distinct events: Female ejaculation described as a small amount of thicker "milky-like" fluid from the prostatic glands (Pastor & Chmel, 2022), and squirting as a larger amount (15–110 mL) of water-like fluid, similar to or identical to urine, expelled from the urethra (Inoue, 2022; Pastor & Chmel, 2022; Rubio-Casillas & Jannini, 2011; Salama et al., 2015). This separation, based on a single study (Rubio-Casillas & Jannini, 2011), was adopted as a new nomenclature in some scientific literature, posing challenges when reviewing earlier works. Given the questionable nature of this distinction, recent studies have combined the terms (Påfs, 2023; Påfs et al., 2024) or utilized the novel term “vaginal squirting” (Hensel et al., 2024). For clarity and consistency, the current study exclusively uses the terminology squirting.

### Women’s Experiences and the Link to Sexual Pleasure and Orgasm

Women’s experiences with squirting vary widely, ranging from feelings of amazement and pride to shame and avoidance (Bullough et al., 1984; Darling et al., 1990; Gilliland, 2009; Hensel et al., 2024; Ladas et al., 1982; Påfs, 2023; Påfs et al., 2024; Wimpissinger et al., 2013). While recent studies in the USA and Sweden generally confirm squirting is perceived as pleasurable (Hensel et al., 2024; Påfs et al., 2024), a common misconception persists: That it is inherently linked to orgasm. Earlier research often conflated the two (e.g., Bullough et al., 1984; Darling et al., 1990; Wimpissinger et al., 2013), but emerging evidence suggests squirting can occur independently of orgasm (Cutillas-Blasco et al., 2024; Gilliland, 2009; Hensel et al., 2024; Påfs, 2023; Påfs et al., 2024). However, how positively women perceive the experience of squirting seems to depend on whether it coincides with orgasm (Påfs et al., 2024).

Furthermore, research suggests that a partner’s responses play a pivotal role in shaping how women experience squirting, particularly during their first encounter (Gilliland, 2009; Påfs, 2023). In a Swedish study, 58% of participants reported having, at some point, wanted to avoid squirting, a desire more common among those who had previously encountered a negative reaction from a partner (Påfs et al., 2024). This influence was also cited in a study from the USA as a reason some women express concern or discomfort about squirting (Hensel et al., 2024). However, a deeper understanding of partners’ responses and how women navigate these interpersonal dynamics is still lacking.

### “Squirting Scripts” within Sexual Interactions Between Women and Men: Context and Theoretical Framework

This study approaches sexuality from a social constructionist perspective, viewing sexual pleasure as shaped by interrelated cultural, interpersonal, and intrapsychic scripts (Gagnon & Simon, 1975; Simon & Gagnon, 1986). While cultural norms inform what is considered appropriate or ideal sexual behavior, interpersonal scripts guide how individuals interact sexually, and the intrapsychic script represents an internal negotiation between personal sexual desires and cultural and interpersonal expectations. These scripts are not static but continuously renegotiated (Gagnon & Simon, 1975; Simon & Gagnon, 1986).

Focusing on the interpersonal dimension of sexual interactions between women and men, this paper emphasizes the reciprocal construction of meaning surrounding squirting. To frame these dynamics, the term squirting scripts is used to refer to narratives that shape expectations and behaviors surrounding the event (Påfs, 2023). While focusing on interactions between women and men, the study draws on gendered norms rather than sexual identity, acknowledging that participants may identify as bisexual, pansexual, or queer.

Through this lens, sexual pleasure carries a gendered meaning where arousal in men is traditionally validated through visible physiological markers like erection and ejaculation. In contrast, women’s bodies have frequently been described as lacking equivalent physical signs (Frith, 2015; Jackson & Scott, 2007). Despite critiques of this androcentric focus, ejaculation remains the definitive symbolic confirmation of orgasm (Salmon et al., 2023; Shor & Chen, 2025). Within sexual interactions between women and men, pleasure is often governed by the coital imperative, the prioritization of penile–vaginal intercourse, and the orgasm imperative, which frames climax as the ultimate measure of sexual success (Braun et al., 2003; Chadwick, 2024; Fahs & Plante, 2017; Frith, 2013; Potts, 2000). These imperatives often transform women’s orgasms into "achievements" or currency used to validate men’s sexual competence and protect their egos (Frith, 2015; Gerhard, 2000; Gill, 2009; Jackson & Scott, 2007; Moran, 2017). The coital script typically dictates a sequence of penetration followed by orgasm for the woman, then the man (or simultaneous climax). A model that often privileges men’s sexuality by positioning women’s orgasm as a precursor to the event’s natural conclusion (Farvid & Braun, 2017; Potts, 2000; Stelzl & Lafrance, 2021). Consequently, orgasms achieved through intercourse are valued more highly than those through manual stimulation or masturbation, a hierarchy reinforced by the language of “real sex” versus “foreplay” (Braun et al., 2003; Frith, 2013; Potts, 2000). This hierarchy is further sustained by a narrow, phallocentric definition of “regular sex” that prioritizes penile stimulation, effectively framing women’s orgasms as “work” or “gender labor” that falls outside the boundaries of the main sexual event (Andrejek et al., 2022). These imperatives have also helped identify the “orgasm gap,” the documented disparity in orgasm frequency between men and women in heterosex encounters, highlighting the unequal outcomes embedded in normative sexual scripts (Andrejek & Fetner, 2019; Mahar et al., 2020).

Faking or exaggerating sexual pleasure has been widely identified as a recurring practice among women, often emerging as a response to the expectation of orgasm as an accomplishment within sexual encounters between women and men (Ford et al., 2023; Frith, 2015; Jackson & Scott, 2007; Kalman, 2008; Thomas et al., 2017). Women report faking for various reasons: to feel “sexually normal,” to avoid making men as sexual partners feel insecure about their sexual performance, or as a strategy to end sex (Chadwick et al., 2019; Fahs, 2014; Ford et al., 2023; Frith, 2015; Jackson & Scott, 2007; Potts, 2000; Thomas et al., 2017). The act creates a dilemma for women, who may feel they are compromising their own values by being dishonest or neglecting their own pleasure, while simultaneously trying to avoid hurting their partner or undermining his sense of masculinity (Chadwick & van Anders, 2017). The act of faking must be subtle enough to be perceived as authentic, something that is learned and negotiated in interaction (Jackson & Scott, 2007). Ultimately, the issue is not simply whether orgasm occurs, but how it is accomplished, embodied, and negotiated through social practice (Jackson & Scott, 2007; Potts, 2000).

### Squirting's Ambiguous Position within the Script

On a cultural level, squirting lacks a singular, universally understood script. In some contexts, such as Rwanda and Ghana, squirting reportedly holds a central, self-evident role in the sexual script, and is an expected part of women’s pleasure (Bizimana, 2010; Fiaveh et al., 2015; Larsen, 2010). In the Global North, however, research on women’s orgasm and sexual pleasure has largely ignored squirting, or dismissed it as a non-signifier (Frith, 2015). At the same time, squirting is highly visible in popular media. Pornography, in particular, uses squirting as “evidence” of women’s pleasure, while erotic short stories utilize the visceral imagery of women’s sexual fluids to arouse sexual excitement (Johnsdotter, 2011; Paasonen, 2006; Ryberg, 2008; Williams, 2016). This portrayal, however, is not without controversy; the UK banned it from pornography in 2014, equating the fluid with urine (Burnett, 2014; Hooton, 2014).

This lack of a coherent cultural narrative has been conceptualized to create a “missing script” (Påfs, 2023), stemming both from widespread unawareness of squirting and from a lack of nuanced understanding (Påfs, 2023). As a result, in the study from Sweden, squirting was perceived as everything from desirable to questionable, with different subcultural scripts emerging. In some communities, such as BDSM or tantra, squirting held a self-evident role and was seen as a sign of pleasure and fulfillment. In contrast, in others, it was considered questionable, mythical, or even an excessive, "hypersexual" norm—the latter mainly due to its association with porn (Påfs, 2023). This association is similarly multifaceted; a study from the USA illustrates that while such depictions in pornography can serve as a source of validation for women who squirt, they can simultaneously create uncertainty among those who do not, leading women to question the validity of their own experiences due to the creation of a new visible norm of orgasm (Frank, 2014). This broad ambivalence highlights the widespread ignorance surrounding this aspect of women’s sexuality, a gap that warrants increased scientific attention.

Within interpersonal sexual scripts between women and men, previous studies have shown that the meaning of squirting is constructed through the negotiation of women’s experiences and the responses of men as partners. A partner’s response, whether real or imagined, seems to play a crucial role in shaping how women navigate this experience during sex and whether they choose to disclose this aspect of their sexuality to others (Fahs, 2017; Gilliland, 2009; Hensel et al., 2024; Påfs, 2023; Påfs et al., 2024). This interpersonal dynamic seems to make the visibility of squirting a charged subject. While it can be interpreted as a powerful sign of pleasure, it is also susceptible to being co-opted by an androcentric framework, an issue this study aims to explore further. Ejaculation has long held symbolic weight as the ultimate marker of climax and power. Potts (2000) identifies a hierarchy in which female ejaculation—phrased as “coming like a guy”—is highly valued for its visible and seemingly incontestable nature. Building on this notion, squirting may be positioned as the counterpart to men’s ejaculation, becoming a desirable “achievement” for men as partners to induce and a tangible “receipt” of pleasure. The emergence of terms like “female cum” further complicates this dynamic by both evoking bodily intensity and positioning women as active sexual subjects, yet this terminology risks reinforcing gendered hierarchies by aligning women’s sexuality too closely with norms traditionally associated with men (Johnsdotter, 2011). As Bell (1991) compellingly argued, women’s capacity to squirt can also be seen as a feminist act of reclaiming the female body, challenging men’s exclusive domain of ejaculation and prompting a re-evaluation of perceived bodily similarities between the sexes. This tension is not only theoretical but also deeply embodied, as the visible wetness of women’s sexual fluids is highly eroticized yet remains underexplored in real-life encounters (Johnsdotter, 2011; Saunders, 2016).

On the intrapsychic level, women’s personal experiences of squirting are shaped by these cultural and interpersonal scripts. For women who were unaware of squirting before it happened, studies indicate that a partner’s reaction influenced their own meaning-making of the experience (Cutillas-Blasco et al., 2024; Gilliland, 2009; Hensel et al., 2024; Påfs, 2023; Påfs et al., 2024). The dissonance between the idea of squirting as a powerful sexual experience and the actual, self-lived experience can cause confusion, disappointment, or embarrassment. This is particularly true since the sensation may not always be orgasmic, in contrast to the semantics embedded in terms like "fountain orgasm" or "squirt orgasm" used in Swedish (Påfs, 2023). Women also contend with negative connotations regarding the fluid’s content (e.g., concern about urine), which can diminish their experience and lead to shameful feelings (Fahs, 2017; Pastor & Chmel, 2017; Salama et al., 2015). This preoccupation with the fluid’s makeup can be viewed as part of a patriarchal suppression of women’s sexuality, as Bell (1991) pointed out. Despite these challenges, some women actively try to rewrite the script, describing squirting as a "sexual superpower" or a "feminist statement," and a visible act of agency that empowers the intrapsychic script (Påfs, 2023).

### Present Study

The present study aims to explore “squirting scripts:” How it is understood and given interpersonal meaning within sexual interactions between women and men, based on cisgender women’s experiences of responses from men as partners. While previous research has examined the invisibility of women’s orgasm and the performative strategies women use to meet normative expectations, squirting introduces a visible bodily response that challenges these narratives. This visibility has led to squirting being interpreted as a powerful and undeniable sign of pleasure (Cutillas-Blasco et al., 2024; Påfs, 2023; Potts, 2000). However, this explicit visibility also renders the experience highly susceptible to partner interpretation, shifting the focus from internal sensation to external validation or scrutiny. Despite its growing presence in popular and pornographic representations (Johnsdotter, 2011; Saunders, 2016), little is known about how squirting is negotiated and interpreted on an interpersonal level. This study addresses that gap. Specifically, it asks: How is squirting understood and given interpersonal meaning within sexual encounters between women and men, based on cisgender women’s experiences of responses from men as partners?

## Method

### Participants

This study draws upon data from in-depth, semi-structured interviews. Participants were recruited via three primary channels: snowball sampling via personal networks; a call for participation on a prominent Swedish sexual health podcast; and a recruitment notice appended to a separate online survey on genital modifications and related practices, sexual pleasure, and squirting (conducted July–September 2019). Interested individuals either initiated contact directly via email or provided their details to be contacted by the author.

During the initial correspondence, the author provided a comprehensive overview of the study and a specific definition of squirting. This screening verified that potential participants’ personal experiences aligned with the study’s focus prior to scheduling an interview. From the pool of eligible respondents, participants were selected specifically to maximize age diversity.

The analytical sample consisted of 16 cisgender women aged 34 to 60. The group reflected diverse relationship statuses, including single, dating, and partnered women within both monogamous and consensually non-monogamous arrangements. Crucially for the aim of this study, all participants had sexual experiences with men, though a few also reported having had sexual experiences with women. All women had experienced squirting with one or several partners.

The participants’ history with squirting varied. Narratives included women who actively sought the experience through exploration, as well as those who discovered it spontaneously later in life, often attributing it to a shift in sexual mindset, meeting a new partner, or experiencing a sexual awakening following the conclusion of a long-term relationship. Frequency and control over the expulsion of fluid also varied widely across the sample.

### Measures and Procedure

The study utilized an exploratory and inductive approach, employing a semi-structured interview guide with open-ended questions. Prior to the main data collection, pilot interviews were conducted to refine the interview guide and inform the study design. The interview guide covered overarching themes of experiences and attitudes toward squirting, responses from others, general aspects of sexuality, and sexual pleasure and practices. This guide was refined throughout the data collection process if new aspects relevant to the research question emerged. To ensure data adequacy, data collection followed an iterative process where emerging data were assessed against previous narratives until topical saturation was met (Guba & Lincoln, 1989).

Prior to the interviews, all participants received verbal and written information about the study’s aim and ethical procedures. Informed consent, including permission for audio recording, was obtained from all subjects. Interviews were conducted by the author either in private settings (face-to-face) or via telephone to facilitate participation from women across Sweden. Interview duration ranged from 45 to 75 min. All data were recorded and transcribed in Swedish; excerpts selected for this manuscript were subsequently translated into English. Participants received no financial compensation. The interviews included in this study were conducted between October 2019 and March 2020.

### Data Analysis

The analysis was guided by the principles of reflexive thematic analysis (Braun & Clarke, 2021). First, the transcribed recordings, as well as field notes that were taken during the interviews, were entered into the software package ATLAS.ti to facilitate the coding process. The sections relevant to the aim of this study were marked and extracted. These were then coded by the author inductively. The coding process was iterative; codes were not fixed at the outset but evolved as the author engaged deeply with the data. The coded sections were re-read to identify patterns of differences and similarities. The codes focused on experiences, expectations, interpretations, and enactment of the sexual response of squirting within sexual encounters between women and men.

When all the transcripts had been coded, and the codes reviewed, preliminary themes were identified to present the variety of narratives in the data. To ensure rigor and validity, the findings and preliminary draft were presented on three different occasions to solicit peer feedback at seminars and scientific conferences by other researchers within the field. Following this feedback, the author finalized the manuscript. During this final interpretive phase, the theoretical framework of sexual scripts was engaged to further theorize the findings, moving the analysis from descriptive themes to the conceptualization of specific “squirting scripts.”

## Results

The narratives concerned responses from men to squirting, drawing directly from participants’ personal experiences. While positive responses were frequently reported, the data revealed complex and various experiences where squirting had been received with negative responses or by strategies of avoidance, or, less commonly, neutral responses. For many, these experiences encompassed a combination of these responses. Four recurring themes were identified to categorize these diverse responses, presented below: Validated, Avoided, Desired, and Coerced.

### Validated: Squirting as an Accepted Sexual Response

A neutral response from a partner was rare in the narratives, yet its presence offered a contrast to the other themes. This validating, unburdened response provided women relief, allowing them to focus less on the act of squirting and more on their own experience. This was particularly evident when it happened for the first time. One woman described being “completely delighted” by the experience yet uncomfortable about the amount of fluid. However, her partner’s calm attitude proved helpful. She recalled: “I was like, ‘Watch out, it’s going to get really wet,’ and he just undramatically responded, ‘That’s fine.’” (#8, age 44). In a similar vein, another woman described a situation where she accidentally squirted a large amount during oral sex. Instead of making a big deal out of it, she described his response as undramatic and lighthearted: “He took it well, like ‘oops, oh well’ [ojdå hoppsan].” (#2, age 55)

Several women expressed it felt crucial that squirting was validated as an ordinary bodily response—nothing more, nothing less—and they narrated this type of validation as essential for their ability to relax in the moment and for squirting to occur. One woman, who had previous experiences of a partner who reacted negatively, emphasized the relief of her current partner’s mature attitude: “My boyfriend now, he can understand that this is a good thing. It’s not better or worse than any other orgasm, and he has more of the attitude like, how exciting that it can be like this.” (#7, age 55)

This validation, particularly when not overemphasized, proved especially pivotal for women who had experienced shame regarding this aspect of their sexual response. In these instances, a partner’s supportiveness or “chill reaction” facilitated their process of acceptance. For one woman, explicit verbal permission from a partner even became a necessary catalyst for releasing the fluid, countering years of suppressed trauma where the fluid had been shamed. She explained: “It is principally based on a partner saying that now it is okay… It has almost become a trigger.” (#13, age 37)

### Avoided: Squirting as an Undesirable Sexual Response

Repeated accounts described squirting as being perceived as undesirable, whether through a partner’s silence, direct expressions of dislike, or active attempts to prevent it. This aversion was sometimes expressed with visceral disgust. One woman recounted an instance where she surprised a partner who became “scared” and physically pulled away because he did not understand what was happening, breaking the intimacy of the moment. She recalled:

“He got wet, and he thought it was pee or something, and said, ‘But what is that?’... I got the impression that he didn’t like it at all, or he kind of pulled away and moved aside and was just, ‘Oh, what is this,’ and got uncomfortable. He didn’t know what it was.” (#7, age 55)

Following this event, she gave “heads-ups” to new partners to prevent similar situations. A few other women also had experiences of a partner who reacted with confusion rather than overt disgust, creating emotional distance. For example, one woman recalled a partner who became visibly bewildered, leading to a “stiff, awkward silence” where he seemed unsure of what had happened or how to react, which in turn made her feel self-conscious (#3, age 45).

While women often noted a variation in partners’ responses—some finding it likable and others not—the primary reasons for perceived avoidance included unawareness of squirting prior to its occurrence, concerns about it being urine, or discomfort with the amount of wetness. The perceived undesirability often stemmed from this wetness and its practical implications. One participant described a response that made her uncomfortable and which she interpreted as dislike, stating:

Well, there have been situations, and it’s a similar situation that has happened a couple of times, where I’ve been with some new man and then I squirted, and they just went completely silent and then “Oh, uh, I should probably get a towel.” And then nothing more. Yes, and then the next time, “Well, now I’ve put out, now I’ve put down a towel, it got wet last time,” and then no further comment. So it has become a bit difficult, or like awkward. They haven’t said outright that it was annoying, but there was just like no comment and then “oh, it gets really wet, I have to get a towel.” Because they didn’t appreciate it. (#5, age 34)

Accounts consistently revealed that the wetness frequently transformed sex into a “project” requiring “post-work” like changing sheets. One participant explicitly described the experience as “cumbersome,” noting the annoyance of having to “clean up afterward” as if “someone had spilled” (#6, age 40). This often led to frustration and the avoidance of spontaneous sexual encounters, lamenting the time commitment for cleanup. The prevalence of narratives focusing on unwanted wet bedsheets and hygiene concerns, such as “who will sleep on the wet spot” (#2, age 55), underscored the tension between spontaneity and the need for preparation. These experiences often led women to try and avoid squirting, or to choose the moment only when fully ready and comfortable with a partner, at least among those who felt they could control it.

### Desired: Squirting as Concrete Proof of Sexual Satisfaction and Sexual Prowess

Squirting was repeatedly explained to be a welcomed experience among the women’s partners, and it seemed to carry intertwined notions of achievement and validation, often creating discrepancies between women’s own subjective experiences and men’s interpretations. While some responses were linked to status, others were described in milder terms of fascination. One participant described her partners’ response as almost innocent, noting: “Most men get delighted, almost what I would perceive as a childish delight, like something happens, and ‘wow, I did that’. It becomes so concrete and tangible, so they seem to perceive it as something cool and fun, and yes, positive” (#3, age 45). Similarly, another woman described her partner’s response as being ignited with enthusiasm. She explained: “He became all fired up and was really happy that he had learned something new. This mythical squirting orgasm, now he knows the secret” (#10, age 51).

The anticipation of squirting as a concrete proof of climax was another common narrative. While some women did experience a climax sensation directly linked to squirting, this correlation did not hold true for others. In narratives equating squirting with orgasm, a distinct terminology emerged: the categorization of orgasms as “wet” or “dry.” A few used this and reported that “wet” orgasms were perceived as more satisfying, both due to personal empowerment and its visibility. Squirting was often cited as desirable precisely because its physical manifestation was seen as irrefutable proof, unlike a “dry” orgasm. One participant explicitly used the term “receipt” to describe how her partner viewed squirting and used the terminology wet/dry, and how “wet” orgasm of squirting did not require further explanation. She said:

He seems to think it represents an orgasm and thinks ‘nice, now you came’... yeah, he thinks it becomes a sort of receipt [...] And maybe if I have a dry orgasm, I explain it, saying that it was good even though I didn’t release anything wet. [...] So anyway, even though we’ve been together for such a long time, I still convey it a bit like that. Because it [the fluid] is so clearly visible. So it’s like it is clearer that I actually came. (#15, age 39)

Accounts of men expressing pride were common, with women reflecting that the experience seemed to boost their partners’ self-esteem and serve as proof that they were “good in bed.” While this positive response could be affirming for the woman, narratives also indicated that some partners interpreted the event specifically as a testament to their own technical skill rather than the woman’s bodily capacity. The focus on squirting as an achievement for men was also evident in narratives where men treated the induction of squirting as a technical skill to be mastered. One participant described a situation where a partner was intent on “learning” how to make her squirt, applying intense effort but lacking emotional connection. She recalled: “He exerted himself so much, and I mostly felt sorry for him” (#9, age 60). In these narratives, the woman’s body appeared to become a vehicle for the man’s ego.

Thus, squirting appeared to have gained a certain status. One participant reflected on the topic’s growing presence in her social circles, noting: “I would claim that it is… a little trendy” (#16, age 49). Another participant described this preoccupation as “sensationalism” (effect-seeking), noting that the emphasis on the visual spectacle of the fluid often overshadowed her own subjective pleasure (#11, age 46). This fixation appeared to transform the act into a “hunt” for some men, creating a dynamic where the aspiration for squirting becomes an externalized desired goal, placing the emphasis on its performative display rather than on the woman’s subjective pleasure.

### Coerced: Squirting as a Pressured Sexual Response

Ideally, positive attention from a partner would be validating, yet several women described how a partner’s intense desire for squirting could transform into pressure. The pursuit of squirting led women to feel compelled or to compromise their personal boundaries in an effort to please their partners and provide them with a squirt. One participant recounted how her partner described witnessing squirting as “a dream” and “the coolest thing he had ever experienced.” While his intention was positive, she admitted that this placed an internal demand on her:

[But] then it became [a worry] for me like: ‘God, does he want me to have this all the time now?’ Because I’ve said I don’t want this to become a performance... but sure, I can feel a little bit beforehand... that now maybe he would think it was nice if it happened. But I really try to let it go. It shouldn’t be like that." (#12, age 43)

A recurring narrative centered on encounters with men who believed they possessed the “right technique” to induce squirting. This perspective often struck women with extensive squirting experience as paradoxical, as they perceived the sexual response as being largely independent of external factors and more centered on their own bodily and mental state. One woman, for instance, observed:

I have seen men post these very detailed manuals on how to proceed, so it also sounds like it’s something complex and something that requires you to do something special to induce it. While I instead have to make an effort to not squirt when I have sex. For me, it’s not about having such a skilled sexual partner. (#5, age 34)

This participant, like several others, sometimes allowed partners to believe it was their achievement, yet knowing from their own experience that it was all about their own mindset.

Experiences of persistent “nagging” were also reported, with narratives of partners trying to persuade them, insisting on exploring certain techniques, or using pornography for inspiration. While intended to enable squirting, participants described the techniques portrayed in such media as problematic. The reliance on external “instructional” content was criticized for promoting standardized, often rough, approach that failed to account for individual bodily variations. In line with this, women recounted specific techniques being “used on them” without sensitivity to their responses. One participant experienced a “burning and uncomfortable sensation,” noting that beyond the physical discomfort, it was an emotional burden when a partner failed to “read” her cues, signifying a misalignment of intentions and a lack of mutual attentiveness. She vividly recalled an ex-partner who specifically aimed for her to squirt, noting how “he was trying to get me somewhere and I was not there yet, he was ahead of me, he was further in the process than I was” (#3, age 45). Despite achieving squirting, she found this experience uncomfortable, emphasizing that mutual attentiveness and pleasure should be the primary goal, not squirting itself.

The notion that actual pleasure got sidelined was mentioned, highlighting how some men viewed squirting as something extraordinary. Partner expectations could create new forms of pressure or worry, particularly for those who were unable to squirt consistently. For instance, one woman (#15, age 39) described an implicit pressure to validate her satisfaction when squirting did not occur, feeling the need to verbally explain that her “dry orgasm” was pleasurable to compensate for the lack of visible proof. Similarly, another woman observed that squirting “has somehow gained this status of being highly desirable” (#14, age 48), noting that this focus on the visual spectacle does not necessarily correlate with the woman’s actual pleasure.

## Discussion

This study’s findings illuminate how squirting is navigated within sexual encounters between women and men, from the perspective of women. The four identified themes—Validated, Avoided, Desired, and Coerced—represent distinct “squirting scripts” and how squirting operates as a complex and contradictory symbolic act at an interpersonal level. These scripts help explain the wide variance of emotional responses—from "amazement and pride to shame and avoidance"—that previous research has documented (e.g., Gilliland, 2009; Hensel et al., 2024; Påfs, 2023). The findings show that squirting, far from being a simple physiological event, carries diverse and often conflicting meanings.

### The “Missing Script” and Its Consequences: Validated and Avoided

The validated and avoided themes directly illustrate the consequences of the “missing script” discussed in the theoretical framework. The avoided theme, where partners reacted with disgust, silence, or confusion (e.g., “Is it pee?”), is an indication of a cultural script that has failed to provide a widespread, normalized understanding of this bodily response. This finding empirically supports the “definitional discord” (Pastor & Chmel, 2022; Salama et al., 2015) identified in the introduction. It illustrates how scientific and cultural ambiguity regarding the fluid’s nature manifests in intimate encounters, where partners react negatively due to the lack of a clear script, often defaulting to stigmatized interpretations of the fluid (mistaking it for urine or incontinence).

This missing script gives rise to an interpersonal script of awkwardness and shame, which in turn can contribute to a negative intrapsychic script for the woman, who may consequently suppress or avoid squirting. This directly reflects previous quantitative findings, where a substantial portion of women reported avoiding squirting specifically due to concerns about negative partner reactions (Hensel et al., 2024; Påfs et al., 2024), and aligns with recent qualitative findings from a Spanish context (Cutillas-Blasco et al., 2024), confirming that negative responses, particularly those involving disgust, profoundly affect women’s relationship with squirting. The practical burdens associated with wetness, often framed as an undesirable “project” requiring cleanup, further highlight how external factors and partner responses transform a potentially pleasurable bodily response into a source of discomfort.

Conversely, the rarity of the Validated (neutral) theme is just as revealing. A calm, undramatic response was powerful because it stood in sharp contrast to the more common polarized scripts of excess or aversion. This neutral interpersonal script seemed crucial for women’s intrapsychic well-being, allowing them to relax and re-frame their experience as a normal bodily function rather than a shameful or extraordinary event. Both themes confirm that in the absence of a clear, established cultural script, the interpersonal negotiation becomes a high-stakes event for women.

### The “Squirting Imperative:” Desired as Irrefutable Proof

The desired theme aligns squirting with the orgasm imperative. While women’s pleasure has traditionally been considered invisible—leading to the practice of faking orgasms to validate male performance (Frith, 2015; Jackson & Scott, 2007)—squirting functions as an incontestable, visible confirmation of this imperative. The current analysis suggests that squirting is being framed as “the ultimate proof” and “the receipt that he’s good in bed,” serving as an antidote to insecurity among men. This dynamic establishes a linguistic and symbolic hierarchy between “wet” and “dry” orgasms, where the former is granted higher credibility due to its undeniable physical manifestation. By providing “irrefutable proof” that cannot be faked, squirting resolves the traditional ambiguity of women’s pleasure but introduces a new pressure.

This creates a paradoxical situation where a woman’s subjective experience of pleasure can be overshadowed by the need to provide this tangible evidence. This aligns with the concept of the orgasm imperative and the pressure to display signs of climax to validate the partner (Chadwick, 2024; Chadwick & van Anders, 2017, 2022). The implied hierarchy, where a “wet” orgasm holds more weight than a “dry” one, reinforces the misconception that squirting is inherently a climax sensation. This highlights a persistent blind spot in the squirting script, where squirting is expected to be an orgasm despite women’s varied experiences (Cutillas-Blasco et al., 2024; Hensel et al., 2024; Påfs et al., 2024).

Consequently, the visibility of the fluid may be transformed into a form of currency: an achievement for men and sign of “sexual prowess,” as seen in narratives of partners treating it as a personal success or an externalized goal. This also indicates squirting may turn into a “hunt.” Thus, squirting as a bodily response may be appropriated as validation for the ego of men, consistent with gendered expectations within sexual encounters between women and men, aligning with research on orgasm as a masculinity achievement (Chadwick & van Anders, 2017).

### The Harmful Side of the Imperative: Coerced Performance

This study’s most novel and troubling finding, the Coerced theme, reveals the potential for harm inherent in the “squirting imperative.” This underscores a critical imbalance in sexual encounters, where a specific outcome—squirting—is prioritized over mutual pleasure and genuine connection. The narratives described scenarios where the pursuit of squirting became a source of pressure, driven by a partner’s excitement, expectations, or misconceptions. This pressure manifested in various ways, from partners asserting a “right technique” (mentioned as “rough”) that sometimes caused discomfort or pain for the woman, to persistent “nagging” that pushed women to compromise their boundaries.

This demonstrates a cultural script, likely fueled by pornography (Saunders, 2016), where a technique is believed to produce a squirt, regardless of the woman’s intrapsychic state (“I was not there yet”). The narratives reveal a dynamic where some women sidelined their bodily integrity or subjective pleasure in favor of a wish or perceived need to “perform” or “provide” a squirt, leading to feelings of stress, inadequacy, or regret. As seen in the findings, this pressure was sometimes subtle and only realized after some distance from the actual event. This echoes broader research on orgasm coercion and its negative impact on women’s sexual enjoyment and autonomy (Chadwick, 2024).

These findings thus suggest a “squirting imperative” has entered the sexual script, where squirting is equated with an extraordinary sexual or orgasmic experience. This intense focus on squirting, similar to how orgasm itself is sometimes valorized or idealized in studies (Chadwick, 2024; Fahs, 2014; Frith, 2013), may reinforce normative scripts of sex and pleasure. By doing so, it can sideline other forms of satisfaction; indeed, the coercive experiences revealed in this study highlight a darker side to this discursive elevation.

Supporting the notion of a squirting imperative, women in this study both expressed feelings of inadequacy when they could not perform a squirt but also questioned the focus on techniques and a “hunt” when they felt it was other aspects in their own body that decided whether it could happen. Squirting thus seems to have become a *performative element*, aligning with concepts of faking orgasm as a response to contradict the discourse of women’s orgasm’s invisibility (Frith, Frith, 2013, 2015; Jackson & Scott, 2007). It further represents a “performance” that men may incorporate within the gendered expectations of sex (Andrejek et al., 2022). The interesting aspect of this performance is that it is created through the gaze of the other, and within the sexual script between women and men, the woman’s actual pleasure and feelings can become secondary to a performance for the gaze of men and potentially as a means to assert masculinity.

### Strengths, Limitations, and Future Direction

A primary strength of this study is its specific focus on the interpersonal negotiation of squirting, addressing a gap in previous research which has largely focused on physiological aspects. By identifying distinct “squirting scripts,” the study offers a theoretical framework for understanding how this bodily response is socially constructed within sexual interactions between women and men. However, limitations exist. The sample consists of self-selected women in Sweden, meaning the findings reflect a specific cultural context and cannot be generalized to the broader population. Furthermore, there is a time lapse between the initial data collection and the publication of this study. While sexual scripts and gendered norms generally evolve gradually, the rapidly increasing visibility of squirting in digital media suggests these dynamics are in flux. This time lapse also limited the feasibility of returning to participants for respondent validation. Nevertheless, the identified themes provide a robust snapshot of the diverse ways squirting was negotiated at the time of the study. To address the issue of generalizability, data for a follow-up quantitative study have been collected to explore the prevalence and distribution of these scripts among a larger sample of women.

### Conclusion

By analyzing women’s accounts of partners’ responses, this study identifies how the bodily occurrence of squirting is not merely a physiological event but a charged symbolic event of social, emotional, and relational meaning-making. By applying the framework of sexual scripts, the analysis demonstrates how this visible response is being navigated in contradictory directions: It is simultaneously a source of shame due to a “missing script” and “definitional discord” (Avoided), a site of normalization and acceptance (Validated), a tangible “irrefutable” marker of the orgasm imperative (Desired), and a new vector for sexual pressure and performance (Coerced).

A key contribution of this study is the identification of a “Squirting Imperative,” shaped by contemporary media representations and internalized gendered expectations of sexual success. Instead of simply challenging the traditional invisibility of women’s pleasure, squirting risks being re-appropriated into existing dynamics of performance and validation. This underscores how women’s sexual experiences remain deeply embedded in, and negotiated through, cultural narratives about gender, pleasure, and control. Ultimately, this study illustrates that squirting may be constructed as a performance and incorporated into the repertoire as a visible marker of achievement. In this light, squirting may function as something that is “done,” serving as a means of demonstrating sexual prowess and reinforcing masculine ideals. This risks prioritizing a goal-oriented display over women’s subjective pleasure.

## Data Availability

The data analyzed during the current study are not publicly available due to the sensitive and personal nature of qualitative interview data, which could compromise participant anonymity and privacy. However, a de-identified version of the data supporting the conclusions of this article may be made available by the author upon reasonable request, in accordance with ethical guidelines and participant consent.
